# Energy estimation methods for positron emission tomography detectors composed of multiple scintillators

**DOI:** 10.1007/s13534-025-00464-w

**Published:** 2025-03-04

**Authors:** Hyeong Seok Shim, Min Jeong Cho, Jae Sung Lee

**Affiliations:** 1 Interdisciplinary Program of Bioengineering, Seoul National University, Seoul, Korea; 2 Integrated Major in Innovative Medical Science, Seoul National University Graduate School, Seoul, Korea; 3 Department of Nuclear Medicine, Seoul National University College of Medicine, Seoul, Korea; 4 Brightonix Imaging Inc., Seoul, Korea

**Keywords:** Positron emission tomography, Scintillation detector, Metascintillator, Phoswich, Energy estimation

## Abstract

The performance and image quality of positron emission tomography (PET) systems can be enhanced by strategically employing multiple different scintillators, particularly those with different decay times. Two cutting-edge PET detector technologies employing different scintillators with different decay times are the phoswich detector and the emerging metascintillator. In PET imaging, accurate and precise energy measurement is important for effectively rejecting scattered gamma-rays and estimating scatter distribution. However, traditional measures of light output, such as amplitude or integration values of photosensor output pulses, cannot accurately indicate the deposit energy of gamma-rays across multiple scintillators. To address these issues, this study explores two methods for energy estimation in PET detectors that employ multiple scintillators. The first method uses pseudo-inverse matrix generated from the unique pulse profile of each crystal, while the second employs an artificial neural network (ANN) to estimate the energy deposited in each crystal. The effectiveness of the proposed methods was experimentally evaluated using three heavy and dense inorganic scintillation crystals (BGO, LGSO, and GAGG) and three fast plastic scintillators (EJ200, EJ224, and EJ232). The energy estimation method employing ANNs consistently demonstrated superior accuracy across all crystal combinations when compared to the approach utilizing the pseudo-inverse matrix. In the pseudo-inverse matrix approach, there is a negligible difference in accuracy when applying integral-based energy labels as opposed to amplitude-based energy labels. On the other hand, in ANN approach, employing integral-based energy labels consistently outperforms the use of amplitude-based energy labels. This study contributes to the advancement of PET detector technology by proposing and evaluating two methods for estimating the energy in the detector using multiple scintillators. The ANN approach appears to be a promising solution for improving the accuracy of energy estimation, addressing challenges posed by mixed scintillation pulses.

## Introduction

Positron emission tomography (PET) stands as a pivotal biomedical imaging tool, enabling the evaluation of the spatiotemporal distribution of positron-emitting radiotracers by detecting two 511 keV gamma-rays emitted from the body following radiotracer injection [5, 26]. PET systems employ indirect radiation detectors consisting of scintillators and photosensors to capture and analyze the gamma-rays, utilizing their energy, position, and timing information to produce high-quality tomographic images [17, 25]. State-of-the-art clinical PET scanners currently utilize a single type of lutetium oxide scintillator characterized by high density, effective atomic number, light yield, and fast decay time. However, the performance and image quality of PET systems can be further enhanced by employing multiple different scintillators, particularly those exhibiting different decay times.

One of the advanced PET detector technologies employing different scintillators with different decay times is the phoswich detector, which is used to determine the depth-of-interaction (DOI) of gamma-rays [3, 6, 8, 11, 12, 27, 32, 37, 41]. DOI measurements improve the uniformity of radial spatial resolution by reducing parallax error occurring at the peripheral field-of-view of ring-type PET systems [9, 17]. DOI information is also useful for enhancing the timing resolution of PET detectors by mitigating the photon transit time variation [7, 33, 36]. In phoswich PET detectors, different scintillators are stacked on photosensors, and DOI is determined through surrogates of decay time, such as the ratio of the integrated charges in the head and tail parts of the scintillation pulse.

Another PET detector technology that utilizes different scintillators with different decay times is the emerging metascintillator [14, 15, 23, 39]. In the metascintillator, thin layers of heavy, dense but slow scintillators are alternated with those of light but fast scintillators at several 100 μm intervals, taking advantages of both materials (high stopping power and fast photon generation) simultaneously.

In PET imaging, accurate and precise energy measurement is important because scattered gamma-rays are rejected and scatter distribution is estimated using energy information. A significant number of events measured in 3D PET are subject to Compton scattering, and substantial inter-crystal scattering events occur in high-resolution PET detectors [19, 22, 34, 38]. The accuracy of scatter correction algorithms based on Monte Carlo or analytic simulation [1, 10, 40] and the first interaction position estimation algorithm of inter-crystal scattering [4, 18, 20, 28] is highly dependent on the energy resolution of the PET detectors. When using a single scintillator type in PET detectors, the number of scintillation photons and the amplitude or integration value of photosensor output pulses are linearly proportional to the gamma-ray energy deposited in the scintillator. However, the slope of this linear relationship between light output and deposit energy varies depending on the scintillator type. Therefore, simple measures of light output, such as the amplitude or integration value of photosensor output pulses, cannot accurately indicate the deposit energy of gamma-rays across multiple scintillators.

In phoswich detectors, single or multiple scattering across different scintillators results in mixed signal waveforms, making energy and DOI estimation difficult through conventional approaches. In metascintillators, recoil electrons generated mainly from heavy and dense scintillators transverse different scintillators because their ionization trajectories are longer than the thickness of alternating scintillator slabs, emitting light photons with different timing properties [15, 39]. To the best of our knowledge, no proper energy estimation method for experimental data has yet been proposed for meta-scintillators generating mixed waveforms. Several methods using GATE simulation or time-correlated single photon counting (TCSPC) set up which offers ground truth of energy have been proposed [13, 24]. However, in actual experiments, it is challenging to obtain ground truth for the energy deposited in each crystal, making it difficult to train the network.

To address these issues, this study explores two methods for energy estimation in PET detectors utilizing multiple scintillators. The first method uses pseudo-inverse matrix composed of the unique pulse profile of each crystal, while the second employs an artificial neural network (ANN) to estimate the energy deposited in each crystal. The effectiveness of the proposed methods was evaluated experimentally using three heavy and dense inorganic scintillation crystals (BGO, LGSO, and GAGG) and three fast plastic scintillators (EJ200, EJ224, and EJ232). In the following sections, we will formulate the problem and present how to solve it using pseudo-inverse and ANN.

## Materials and methods

### Problem formulation

The scintillation pulse from a single type scintillator can be formulated as following equation:

1 $$ f_{scintillation} \left( t \right) = \frac{E \times LY}{{\tau_{{{\text{decay}}}} - \tau_{{{\text{rise}}}} }}\left( {{\text{e}}^{{ - \frac{{\text{t}}}{{{\uptau }_{{{\text{decay}}}} }}}} - {\text{e}}^{{ - \frac{{\text{t}}}{{{\uptau }_{{{\text{rise}}}} }}}} } \right), $$

where *E* is deposited energy of gamma-ray, *LY* light yield, *t* time, *τ*_decay_ decay time constant, and *τ*_rise_ rising time constant.

Because all parameters except for *E* and *t* are constant in this formula, the peak amplitude and time integral of the scintillation pulse are linearly proportional to the energy *E* deposited in scintillator, thus allowing them to be used as energy surrogates.

Meanwhile, the mixed scintillation pulse from two scintillators can be formulated as following equation:

2 $$\begin{aligned} f_{mixed - scintillation} \left( t \right) &= \frac{{E_{1} \times LY_{1} }}{{\tau_{{{\text{decay}}1}} - \tau_{{{\text{rise}}1}} }}\left( {{\text{e}}^{{ - \frac{{\text{t}}}{{{\uptau }_{{{\text{decay}}1}} }}}} - e^{{ - \frac{{\text{t}}}{{{\uptau }_{{{\text{rise}}1}} }}}} } \right) \\ &\quad+ \frac{{\left( {E_{{{\text{total}}}} - E_{1} } \right) \times LY_{2} }}{{\tau_{{{\text{decay}}2}} - \tau_{{{\text{rise}}2}} }}\left( {{\text{e}}^{{ - \frac{{\text{t}}}{{{\uptau }_{{{\text{decay}}2}} }}}} - {\text{e}}^{{ - \frac{{\text{t}}}{{{\uptau }_{{{\text{rise}}2}} }}}} } \right), \end{aligned}$$

where *E*_total_ is deposited energy of gamma-ray, *E*_1_ is deposited energy on crystal 1, *E*_2_ is deposited energy on crystal 2, *LY*_1_ is the light yield of crystal 1, *LY*_2_ is the light yield of crystal 2, *t* time, *τ*_decay1_ decay time constant of crystal 1,, *τ*_decay2_ decay time constant of crystal 2, and *τ*_rise1_ rising time constant of crystal 1,* τ*_rise2_ rising time constant of crystal 2.

Because each scintillator has its own unique light yield and timing properties, the peak amplitude and time integral of the mixed pulses do not correspond to a single value of total deposit energy across the scintillators. Therefore, these conventional energy surrogates are inappropriate for estimating the energy of gamma-rays from the mixed pulses. For more accurate energy assessment, it is necessary to separate the mixed pulse into two bi-exponential components and estimate the energy of each component based on unique property of each scintillator. The energy of two components can then be summed to obtain the total energy of gamma-ray interaction with scintillators.

As mentioned previously, two different methods using either pseudo-inverse matrix or ANN can be used to estimate the energy of each component. We generated mixed pulses by combining the scintillation pulses generated from each scintillator and estimated the energy from the mixed pulses using the two methods.

### Pseudo inverse matrix method

The mixed signal can be expressed in matrix form as follows:

3 $$ y = {\varvec{A}} \times X + N $$

*y* ∈ ℝ^1024×1^: The signal obtained by summing the signal pulses from two crystals; ***A*** ∈ ℝ^1024×2^: Each column vector of ***A*** is the normalized signal pulse profile of each crystal; *X* ∈ ℝ^2×1^: Each row of *X* is the energy of each crystal; N ∈ ℝ^1024×1^: zero-mean additive white Gaussian noise (AWGN).

Because AWGN was assumed, the maximum-likelihood solution of Eq. (3) can be directly calculated using the pseudo-inverse matrix as follows:

4 $$ \hat{x} = \left( {{\varvec{A}}^{{\mathbf{T}}} {\varvec{A}}} \right)^{{ - {\mathbf{1}}}} {\varvec{A}}^{{\mathbf{T}}} {\varvec{y}} $$

The normalized pulse profile for each crystal was obtained by taking the average of 5000 pulses from each crystal which were normalized by their peak amplitudes. The integral and amplitude value were utilized as the energy value. The accuracy of energy estimation was assessed by the average *R*^2^ value (coefficient of determination) between the ground true and estimated energy labels.

### Artificial neural network

We utilized a dataset composed of 5000 to 10,000 events per scintillator combination and divided it into training and validation data in a 4:1 ratio. To ensure effective training, we divided input signal by 1000 and the labels by 10. The ANN model used for energy estimation was a multi-layer perceptron with two hidden layers. The number of nodes in each layer was 256, and ReLU activation function was used. The input was a 1024 × 1 vector representing the sum of two pulses, and the output was a 2 × 1 vector representing the energy labels of the two pulses. The accuracy of energy estimation was also assessed by the average *R*^2^ value between the true and estimated energy labels.

### Experimental setup

The size of scintillators (BGO, LGSO, GAGG, EJ200, EJ228, and EJ232) used in this study was commonly 3 mm × 3 mm × 20 mm. All sides of the scintillators were polished and wrapped with ESR reflectors. The main characteristics of the scintillators are summarized in Table 1.

**Table 1:** Main characteristics of the scintillators used in the experiments

	Dense scintillation crystals			Fast plastic scintillators		
	BGO	LGSO	GAGG	EJ200	EJ228	EJ232
Density (g/cm^3^)	7.13	7.15	6.63	1.02	1.02	1.02
Peak emission wavelength (nm)	480	430	530	425	391	370
Light yield (photons/MeV)	8500	28,000	42,000	10,000	10,200	8400
Decay time (ns)	317	40	90	2.1	1.4	1.6

**Fig. 1:**
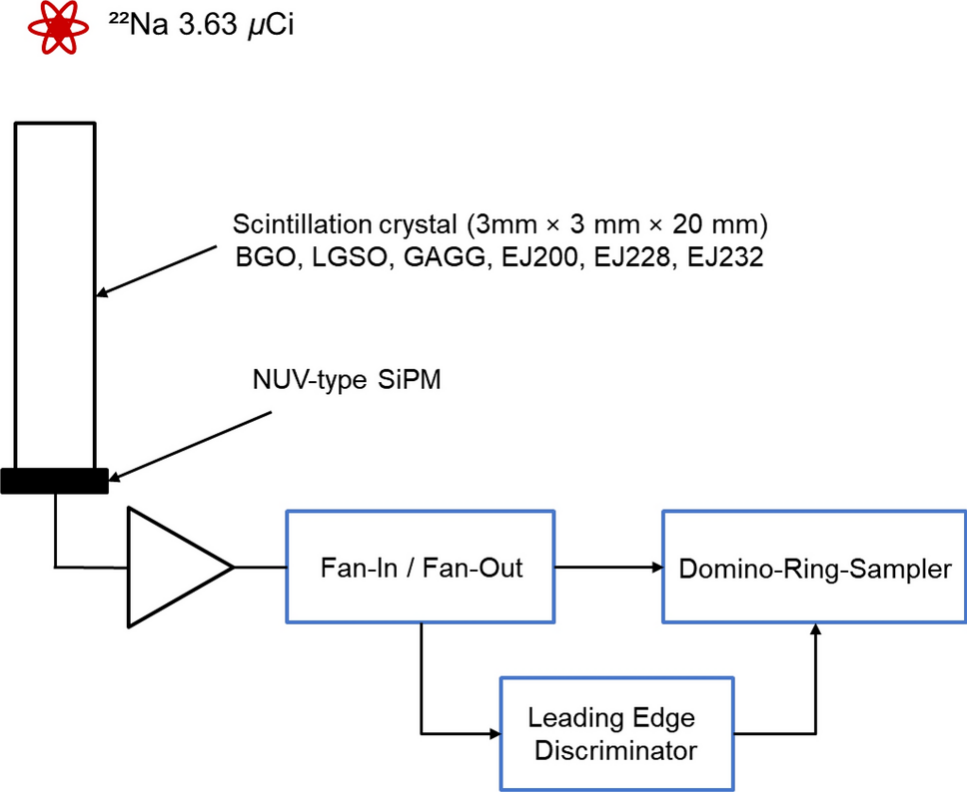
Experimental setup

A total of 12 combinations were used in the experiment. Among these combinations, three were composed of dense crystals: BGO-GAGG, BGO-LGSO, and LGSO-GAGG. The remaining nine combinations consisted of one dense crystal and one fast crystal. The scintillators were coupled with NUV-type silicon photomultipliers (AFBR-S4N44C013; Broadcom, USA) with a pitch size of 3.72 mm × 3.72 mm. The overvoltage was kept constant at 3 V, and the experiments were conducted at 25 °C. The set-up was shielded by placing inside the temperature controlled chamber, and was set to 20 ℃. The distance between the point source and the detector was 10 cm. All data were 1 μs long and obtained using a Domino-Ring-Sampler (DT5742B, Caen, Italy) with 1 GHz sampling rate. SiPM signals were split using a Fan-In/Fan-Out module (N625, Caen, Italy) and trigger was generated using a leading-edge discriminator (N840, Caen, Italy) The circuit diagram is shown in Fig. 1.

## Results

### Pseudo inverse matrix method

**Fig. 2:**
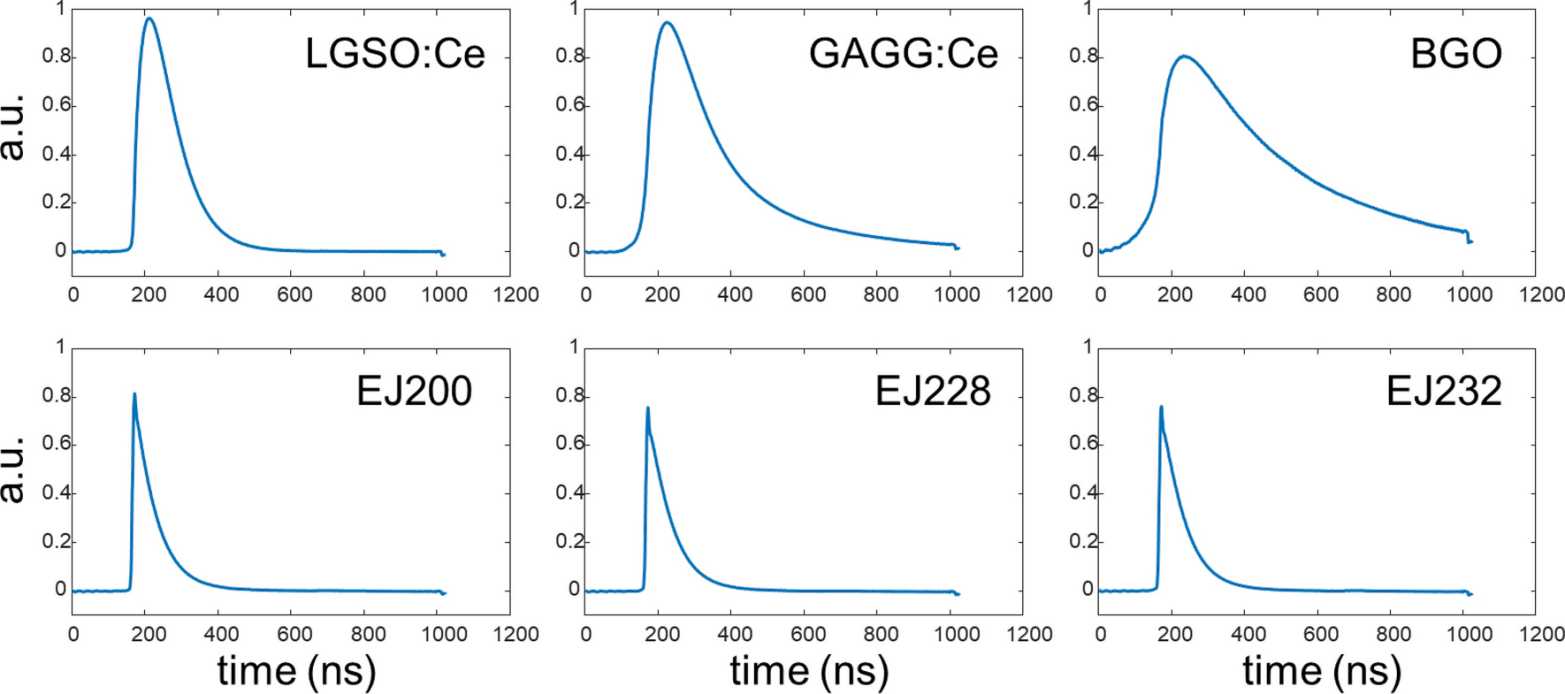
Signal profile of each crystal

**Fig. 3:**
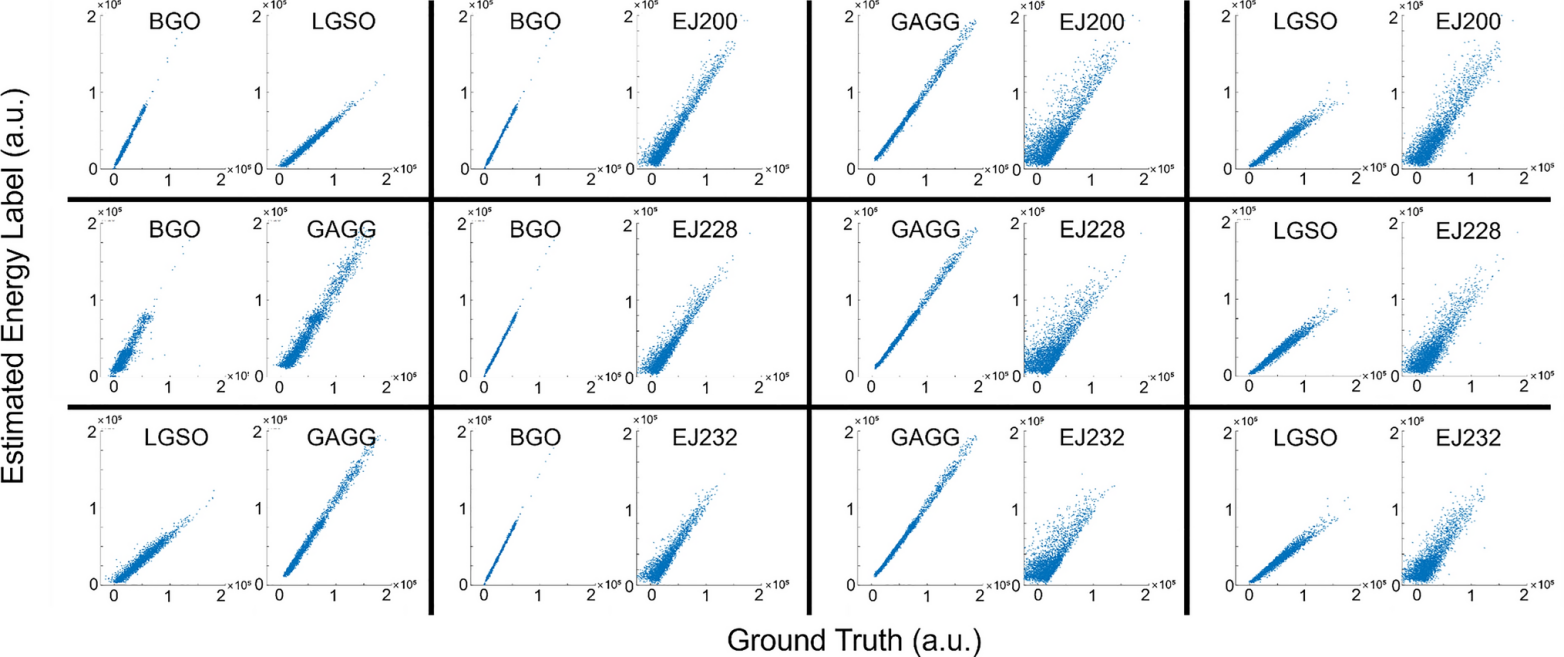
Scatter plot between the ground truth and the estimated time integral of scintillation pulse obtained by computing pseudo-inverse matrix

**Fig. 4:**
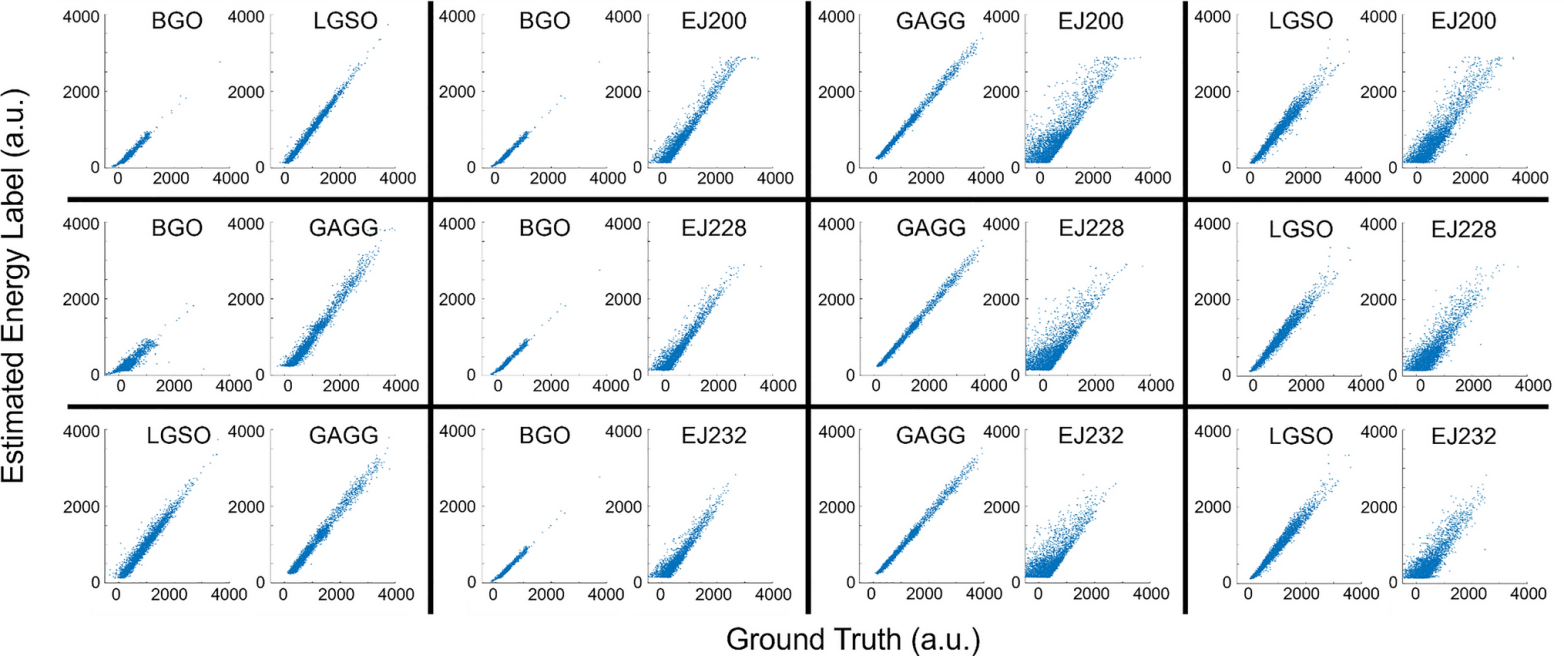
Scatter plot between the ground truth and the estimated amplitude of scintillation pulse obtained by computing pseudo-inverse matrix

Figure 2 shows the signal profile of each crystal, and Figs. 3 and 4 show the scatter plots between the ground truth and the estimated energy labels (Fig. 3: integral, Fig. 4: amplitude) obtained by computing the pseudo-inverse matrix for the combined signal profiles. The *R*^2^ values between the ground truth and estimated energy labels for the combinations of dense crystals are summarized in Table 2. In Tables 3 and 4, the *R*^2^ values between the ground truth and estimated energy labels for the combinations of one dense and one fast scintillator are summarized (Table 3: integral, Table 4: amplitude).

**Table 2:** *R*^2^ value between the ground truth and the estimated energy labels using pseudo inverse matrix for the combinations of dense crystals

Energy label	BGO/LGSO	BGO/GAGG	LGSO/GAGG
Integral	0.99/0.98	0.78/0.92	0.91/0.96
Amplitude	0.95/0.99	0.72/0.95	0.91/0.99

**Table 3:** *R*^2^ value between the ground truth and the estimated time integral of scintillation pulse obtained by computing pseudo-inverse matrix (dense/fast scintillation combinations)

	BGO	GAGG	LGSO
EJ200	0.99/0.93	0.99/0.68	0.96/0.81
EJ228	0.99/0.88	0.99/0.53	0.97/0.72
EJ232	0.99/0.86	0.99/0.48	0.97/0.68

**Table 4:** *R*^2^ value between the ground truth and the estimated amplitude of scintillation pulse obtained by computing pseudo-inverse matrix (dense/fast scintillation combinations)

	BGO	GAGG	LGSO
EJ200	0.97/0.93	0.99/0.68	0.96/0.81
EJ228	0.97/0.88	0.99/0.54	0.97/0.72
EJ232	0.97/0.86	0.99/0.48	0.97/0.69

Among the combinations of dense crystals, the BGO—LGSO combination achieved the best results in both cases when using amplitude and integral as energy label. Among the combination of one dense crystal and one fast crystal, the BGO—EJ200 combination achieved the best results.

### Artificial neural network method

**Fig. 5:**
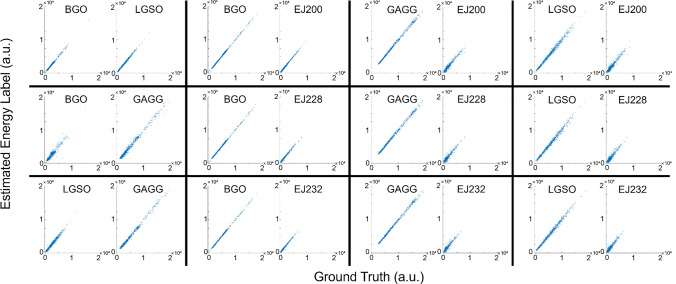
Scatter plot between the ground truth and the estimated time integral of scintillation pulse obtained using ANN

**Fig. 6:**
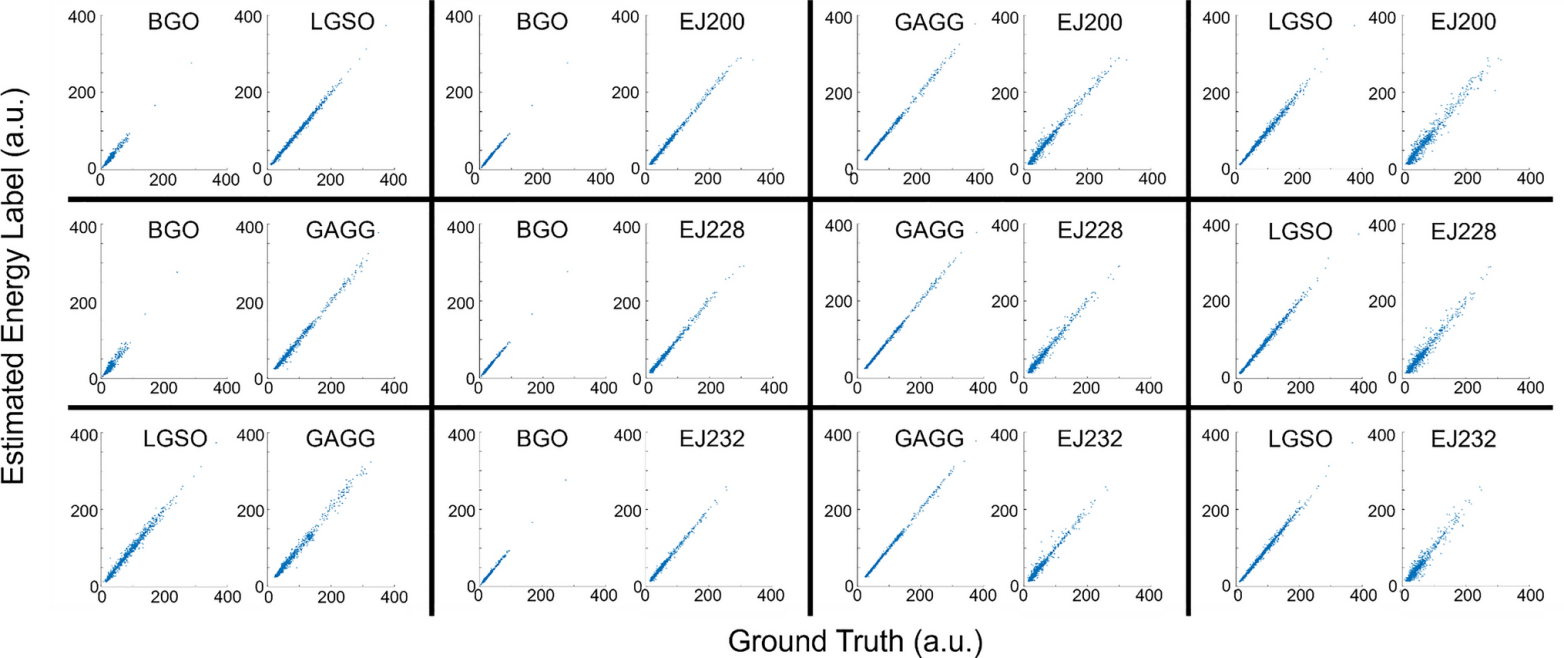
Scatter plot between the ground truth and the estimated amplitude of scintillation pulse obtained using ANN

Figures 5 and 6 show the scatter plots between the ground truth and the estimated energy labels (Fig. 5: time integral, Fig. 6: amplitude) obtained using ANN applied to the combined signal profiles. The *R*^2^ values between the ground truth and estimated energy labels for the combinations of dense crystals are summarized in Table 5. In Tables 6 and 7, the *R*^2^ values between the ground truth and estimated energy labels for the combinations of one dense and one fast scintillator are summarized (Table 6: time integral, Table 7: amplitude).

**Table 5:** *R*^2^ value between the ground truth and the estimated energy labels using ANN for the combinations of dense crystals

Energy label	BGO/LGSO	BGO/GAGG	LGSO/GAGG
Integral	0.99/0.99	0.93/0.99	0.97/0.99
Amplitude	0.96/0.99	0.92/0.99	0.98/0.99

**Table 6:** *R*^2^ value between the ground truth and the estimated time integral of scintillation pulse obtained using ANN (dense/fast scintillation combinations)

	BGO	GAGG	LGSO
EJ200	0.99/0.99	0.99/0.98	0.99/0.96
EJ228	0.99/0.99	0.99/0.98	0.99/0.95
EJ232	0.99/0.99	0.99/0.97	0.99/0.94

**Table 7:** *R*^2^ value between the ground truth and the estimated amplitude of scintillation pulse obtained using ANN (dense/fast scintillation combinations)

	BGO	GAGG	LGSO
EJ200	0.99/0.99	0.99/0.96	0.99/0.94
EJ228	0.99/0.99	0.99/0.93	0.99/0.91
EJ232	0.99/0.98	0.99/0.92	0.99/0.91

Among the combinations of dense crystals, the BGO—LGSO combination performed the best when using integral as energy label while the LGSO—GAGG combination yielded the best results when using amplitude as energy label. Among the combination of one dense crystal and one fast crystal, the BGO—EJ200 combination achieved the best results in both cases when using amplitude and integral as energy label.

## Discussion and conclusion

Introducing multiple scintillators can improve system performance and image quality in PET scanners. The use of phoswich detectors and emerging metascintilators, each employing scintillators with different decay times, makes accurate energy measurements difficult in PET scans. Traditional energy measures, such as peak amplitude and time integration of scintillation pulses, are inadequate when dealing with mixed pulses of different scintillators, so advanced methods for energy estimation are needed. Therefore, in this study, we explored two methods for energy estimation of PET detectors utilizing multiple scintillators: pseudo-inverse matrix and ANN approaches. The goal of this paper is to evaluate two energy estimation methods for scenarios where a single gamma ray interacts with two scintillation crystals. The pseudo-inverse matrix method utilizes the known pulse profiles of individual crystals to provide a simple and interpretable solution. On the other hand, ANN approaches offer flexibility and adaptability, potentially accommodating more complex relationships between input signals and energy labels.

The energy estimation method employing ANNs consistently demonstrated superior accuracy across all crystal combinations when compared to the approach utilizing the pseudo-inverse matrix. This notable performance gap would arise from the inherent limitation of the pseudo-inverse matrix method in effectively compensating for time-walk. More specifically, the pseudo-inverse matrix method has limitation in rectifying instances of time-walk, as evidenced by the signal profiles shown in Fig. 2, where peak amplitudes deviate from the ideal value of 1. Time-walk, in this context, is attributed to the process of generating the trigger signals. As illustrated in Fig. 1, the trigger signals were generated through leading-edge discrimination. To mitigate the time-walk effects, it would be necessary to minimize the threshold level while maintaining it above the electrical noise level.

In the pseudo-inverse matrix approach, there is a negligible difference in accuracy when applying integral-based energy labels as opposed to amplitude-based energy labels (refer to Tables 3 and 4). On the other hand, in ANN approach, employing integral-based energy labels consistently outperforms the use of amplitude-based energy labels. While energy labels theoretically should yield identical values for integral and amplitude in an ideal, noise-free scenario, the robustness of the integral against noise is beyond doubt. Therefore, using integrals as energy labels appears to be a better strategy to achieve improved accuracy compared to using amplitudes as energy labels, especially in the presence of noise.

In conclusion, this study contributes to the development of PET detector technology by proposing and evaluating two methods for estimating the energy of the detector using multiple scintillators. The ANN approach appears to be a promising method to improve the accuracy of energy estimation, addressing challenges posed by mixed scintillation pulses. The experimental setup, involving combinations of heavy and dense inorganic and fast plastic scintillators, provided a diverse dataset for rigorous evaluation. The consistency of results across different combinations suggests the robustness of the proposed methods. Further research may explore the scalability and adaptability of these methods to different scintillator combinations and PET system configurations. We hope that this research, along with various recent artificial intelligence and machine learning studies to improve not only various biomedical image processing and analysis techniques [16, 29–31] but also the performance of PET detectors [2, 21, 35], will ultimately contribute to improving PET system performance.

## References

[1] Accorsi R, Adam LE, Werner ME, Karp JS. Optimization of a fully 3D single scatter simulation algorithm for 3D PET. Phys Med Biol. 2004;49:2577–98. 15272675 10.1088/0031-9155/49/12/008

[2] Berg E, Cherry SR. Using convolutional neural networks to estimate time-of-flight from PET detector waveforms. Phys Med Biol. 2018;63:02LIT. 10.1088/1361-6560/aa9dc5 PMC5784837 29182151

[3] Chandrikamohan P, DeVol TA. Comparison of pulse shape discrimination methods for phoswich and CsI: Tl detectors. IEEE Trans Nucl Sci. 2007;54:398–403.

[4] Comanor K, Virador P, Moses W. Algorithms to identify detector Compton scatter in PET modules. IEEE Trans Nucl Sci. 1996;43:2213–8.

[5] Dhawan V, Niethammer MH, Lesser ML, Pappas KN, Hellman M, Fitzpatrick TM, Bjelke D, Singh J, Quatarolo LM, Choi YY, Oh A, Eidelberg D, Chaly T. Prospective F-18 FDOPA PET imaging study in human PD. Nucl Med Mol Imaging. 2022;56:147–57. 35607632 10.1007/s13139-022-00748-4 PMC9123108

[6] Gu Z, Prout DL, Silverman RW, Herman H, Dooraghi A, Chatziioannou AF. A detector with crystal scatter identification capability for high sensitivity and high spatial resolution PET imaging. IEEE Trans Nucl Sci. 2015;62:740–7. 26478600 10.1109/TNS.2015.2408333 PMC4608445

[7] Guo L, Tian J, Chen P, Derenzo S, Choong W-S. Improving timing performance of double-ended readout in TOF-PET detectors. J Instrum. 2020;15:P01003. 33273960 10.1088/1748-0221/15/01/p01003 PMC7710007

[8] Hong SJ, Kwon SI, Ito M, Lee GS, Sim K-S, Park KS, Rhee JT, Lee JS. Concept verification of three-layer detectors for small animal. PET IEEE Trans Nucl Sci. 2008;55:912–7.

[9] Ito M, Hong SJ, Lee JS. Positron emission tomography (PET) detectors with depth-of-interaction (DOI) capability. Biomed Eng Lett. 2011;1:70–81.

[10] John MO. Model-based scatter correction for fully 3D PET. Phys Med Biol. 1996;41:153. 8685253 10.1088/0031-9155/41/1/012

[11] Jung JH, Choi Y, Chung YH, Devroede O, Krieguer M, Bruyndonckx P, Tavernier S. Optimization of LSO/LuYAP phoswich detector for small animal PET. Nucl Instrum Methods Phys Res A. 2007;571:669–75.

[12] Karp JS, Daube-Witherspoon ME. Depth-of-interaction determination in NaI (Tl) and BGO scintillation crystals using a temperature gradient. Nucl Instrum Methods Phys Res A. 1987;260:509–17.

[13] Konstantinou G, Zhang L, Bonifacio D, Latella R, Benlloch JM, Gonzalez AJ. Semi-monolithic meta-scintillator simulation proof-of-concept, combining accurate and TOF. IEEE Trans Radiat Plasma Med Sci. 2024;8:482–92.

[14] Konstantinou G, Latella R, Moliner L, Zhang L, Benlloch JM, Gonzalez AJ, Lecoq P. A proof-of-concept of cross-luminescent metascintillators: testing results on a BGO:BaF2 metapixel. Phys Med Biol. 2023;68:025018. 10.1088/1361-6560/acac5f 36595320

[15] Konstantinou G, Lecoq P, Benlloch JM, Gonzalez AJ. Metascintillators for ultrafast gamma detectors: a review of current state and future perspectives. IEEE Trans Radiat Plasma Med Sci. 2022;6:5–15.

[16] Lee JS. A review of deep-learning-based approaches for attenuation correction in positron emission tomography. IEEE Trans Radiat Plasma Med Sci. 2021;5:160–84.

[17] Lee JS, Lee MS. Advancements in positron emission tomography detectors: from silicon photomultiplier technology to artificial intelligence applications. PET Clinics. 2024;19:1–24. 37802675 10.1016/j.cpet.2023.06.003

[18] Lee MS, Kang SK, Lee JS. Novel inter-crystal scattering event identification method for PET detectors. Phys Med Biol. 2018;63: 115015. 29658493 10.1088/1361-6560/aabe3a

[19] Lee S, Kim KY, Lee MS, Lee JS. Recovery of inter-detector and inter-crystal scattering in brain PET based on LSO and GAGG crystals. Phys Med Biol. 2020;65: 195005. 32575086 10.1088/1361-6560/ab9f5c

[20] Lee S, Lee JS. Inter-crystal scattering recovery of light-sharing PET detectors using convolutional neural networks. Phys Med Biol. 2021;66: 185004. 10.1088/1361-6560/ac215d 34438380

[21] Lee S, Lee JS. Experimental evaluation of convolutional neural network-based inter-crystal scattering recovery for high-resolution PET detectors. Phys Med Biol. 2023;68: 095017. 10.1088/1361-6560/accacb 37019126

[22] Lee S, Lee MS, Kim KY, Lee JS. Systematic study on factors influencing the performance of interdetector scatter recovery in small-animal PET. Med Phys. 2018;45:3551–62. 10.1002/mp.13020 29851131

[23] Pagano F, Kratochwil N, Salomoni M, Pizzichemi M, Paganoni M, Auffray E. Advances in heterostructured scintillators: toward a new generation of detectors for TOF-PET. Phys Med Biol. 2022;67: 135010. 10.1088/1361-6560/ac72ee 35609611

[24] Pagano F, Kratochwil N, Martinazzoli L, Lowis C, Paganoni M, Pizzichemi M. Modeling scintillation kinetics and coincidence time resolution in heterostructured scintillators. IEEE Trans Nucl Sci. 2023;70:2630–7.

[25] Park H, Yi M, Lee JS. Silicon photomultiplier signal readout and multiplexing techniques for positron emission tomography: a review. Biomed Eng Lett. 2022;12:263–83. 35892029 10.1007/s13534-022-00234-y PMC9308856

[26] Peng F. Recent advances in cancer imaging with 64CuCl2 PET/CT. Nucl Med Mol Imaging. 2022;56:80–5. 35464672 10.1007/s13139-022-00738-6 PMC8976861

[27] Prout DL, Gu Z, Shustef M, Chatziioannou AF. A digital phoswich detector using time-over-threshold for depth of interaction in PET. Phys Med Biol. 2020;65: 245017. 33202397 10.1088/1361-6560/abcb21 PMC8382115

[28] Rafecas M, Böning G, Pichler B, Lorenz E, Schwaiger M, Ziegler S. Inter-crystal scatter in a dual layer, high resolution LSO-APD positron emission tomograph. Phys Med Biol. 2003;48:821–48. 12701889 10.1088/0031-9155/48/7/302

[29] Rajendran P, Sharma A, Pramanik M. Photoacoustic imaging aided with deep learning: a review. Biomed Eng Lett. 2022;12:155–73. 35529338 10.1007/s13534-021-00210-y PMC9046497

[30] Rao D, Prakashin K, Singh R. Automated segmentation of the larynx on computed tomography images: a review. Biomed Eng Lett. 2022;12:175–83. 35529346 10.1007/s13534-022-00221-3 PMC9046475

[31] Reader AJ, Corda G, Mehranian A, da Costa-Luis C, Ellis S, Schnabel JA. Deep learning for PET image reconstruction. IEEE Trans Radiat Plasma Med Sci. 2020;5:1–25. 10.1109/TRPMS.2020.3004408 PMC7610859 34056150

[32] Seidel J, Vaquero JJ, Siegel S, Gandler WR, Green MV. Depth identification accuracy of a three layer phoswich PET detector module. IEEE Trans Nucl Sci. 1999;46:485–90.

[33] Seo M, Park H, Lee S, Ko GB, Lee JS. Depth-of-interaction positron emission tomography detector with 45° tilted silicon photomultipliers using dual-ended signal readout. Med Phys. 2023;50:4112–21. 36907664 10.1002/mp.16355

[34] Shao Y, Cherry SR, Siegel S, Silverman RW. A study of inter-crystal scatter in small scintillator arrays designed for high resolution PET imaging. IEEE Trans Nucl Sci. 1996;43:1938–44.

[35] Shim H, Bae S, Lee S, Lee J. Inter-crystal scattering event identification using a novel silicon photomultiplier signal multiplexing method. Phys Med Biol. 2023;68: 115008. 10.1088/1361-6560/acd163 37116513

[36] Spanoudaki VC, Levin C. Investigating the temporal resolution limits of scintillation detection from pixellated elements: comparison between experiment and simulation. Phys Med Biol. 2011;56:735. 21239845 10.1088/0031-9155/56/3/013

[37] Streun M, Brandenburg G, Larue H, Saleh H, Zimmermann E, Ziemons K, Halling H. Pulse shape discrimination of LSO and LuYAP scintillators for depth of interaction detection in PET. IEEE Trans Nucl Sci. 2003;50:344–7.

[38] Thompson C. The effect of collimation on scatter fraction in multi-slice PET. IEEE Trans Nucl Sci. 1988;35:598–602.

[39] Turtos RM, Gundacker S, Auffray E, Lecoq P. Towards a metamaterial approach for fast timing in PET: experimental proof-of-concept. Phys Med Biol. 2019;64: 185018. 30978716 10.1088/1361-6560/ab18b3

[40] Watson CC. New, faster, image-based scatter correction for 3D PET. IEEE Trans Nucl Sci. 2000;47:1587–94.

[41] Yamamoto S, Kobayashi T, Okumura S, Yeom JY. Timing performance measurements of Si-PM-based LGSO phoswich detectors. Nucl Instrum Methods Phys Res A. 2016;821:101–8.

